# Activating Technology for Connected Health in Cancer: Protocol for a Research and Training Program

**DOI:** 10.2196/resprot.8900

**Published:** 2018-01-24

**Authors:** Nicola Mountford, Enrique Dorronzoro Zubiete, Threase Kessie, Begonya Garcia-Zapirain, Roberto Nuño-Solinís, David Coyle, Kristin B Munksgaard, Luis Fernandez-Luque, Octavio Rivera Romero, Matilde Mora Fernandez, Pedro Valero Jimenez, Ailish Daly, Ruth Whelan, Brian Caulfield

**Affiliations:** ^1^ Insight Centre University College Dublin Dublin Ireland; ^2^ School of Business University College Dublin Dublin Ireland; ^3^ Department of Electronic Technology Universidad de Sevilla Seville Spain; ^4^ University of Deusto Bilbao Spain; ^5^ School of Computer Science University College Dublin Dublin Ireland; ^6^ University of Southern Denmark Kolding Denmark; ^7^ Salumedia Seville Spain; ^8^ Universidad de Sevilla Seville Spain; ^9^ Oncoavanze Seville Spain; ^10^ Beacon Hospital Dublin Ireland; ^11^ School of Public Health, Physiotherapy and Sports Science University College Dublin Dublin Ireland

**Keywords:** eHealth, mHealth, consumer health informatics, cancer, cancer rehabilitation

## Abstract

**Background:**

As cancer survival rates increase, the challenge of ensuring that cancer survivors reclaim their quality of life (QoL) becomes more important. This paper outlines the research element of a research and training program that is designed to do just that.

**Objective:**

Bridging sectors, disciplines, and geographies, it brings together eight PhD projects and students from across Europe to identify the underlying barriers, test different technology-enabled rehabilitative approaches, propose a model to optimize the patient pathways, and examine the business models that might underpin a sustainable approach to cancer survivor reintegration using technology.

**Methods:**

The program, funded under the European Union's Horizon 2020 research and innovation program under the Marie Sklodowska-Curie grant agreement No 722012, includes deep disciplinary PhD projects, intersectoral and international secondments, interdisciplinary plenary training schools, and virtual subject-specific education modules.

**Results:**

The 8 students have now been recruited and are at the early stages of their projects.

**Conclusions:**

CATCH will provide a comprehensive training and research program by embracing all key elements—technical, social, and economic sciences—required to produce researchers and project outcomes that are capable of meeting existing and future needs in cancer rehabilitation.

## Introduction

The world of medical science has been waging a war on cancer for decades—a war which is slowly but surely being won. But the victories have been seen on the battlefields for survival, with the goal being life itself, through early detection and aggressive treatments. In short, the mantra to-date has been simple: “save the life, kill the cancer.” There were just over 3.4 million new cases of cancer (excluding non-melanoma skin cancers) in Europe in 2012. Mathers and Loncar estimate that by 2030, 20 million people in the World Health Organization European Region will be living with cancer diagnosed five years previously [1].

Although the number of cases of cancer is increasing, so are the survival rates of patients in Europe [2] and worldwide [3]. However, cancer and its treatments cause many physical and psychological symptoms and side effects. Physical symptoms include loss of power and function in limbs, muscle wasting, chronic fatigue, and loss of appetite, while psychological symptoms range from depression, anxiety associated with uncertainty, poor body image, and loss of intimacy in relationships [4]. All contribute to reducing the overall QoL (Quality Of Life)[5], the very thing that we are seeking to maximize within cancer-recovery cohorts (see studies in breast cancer [6], [7] and prostate cancer [8],[9]). We know that increased physical exercise within such cohorts has a positive impact on QoL and contributes to the prevention of recurrence [10]–[11]. In addition, increasing physical activity can alleviate long term side effects of new cancer treatment, such as fatigue, offering the “potential to reinstall structure in everyday life.” [12]

Cancer: Activating Technology for Connected Health (CATCH) seeks to maximize these restorative powers of physical activity in cancer cohorts while understanding that there are both physical and mental barriers that survivors encounter when seeking to exercise. Therefore, the core objective of the study is to activate technology in bridging the gap between cancer survivors’ depleted physical and emotional state and their ultimate ability to return to a fully functional societal role through technology-supported physical exercise.

**Figure 1:**
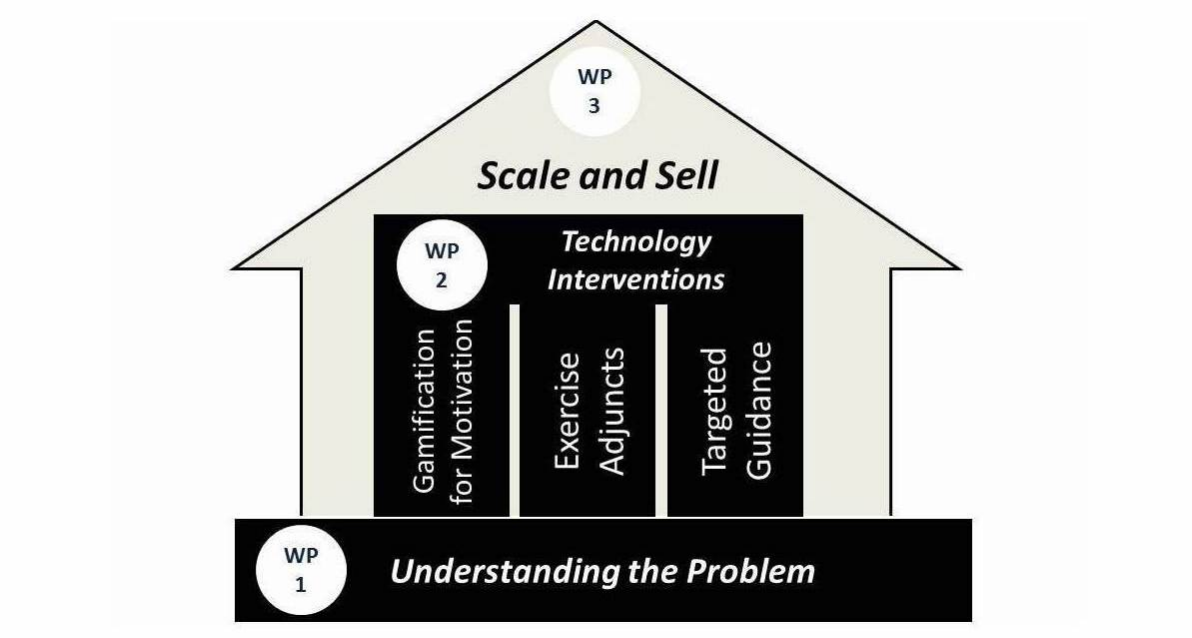
The Cancer: Activating Technology for Connected Health (CATCH) Research Program.

To achieve the core objective there are three sub-objectives: 1) to understand the nature of the gap (from the patient perspective), 2) to design and test technology-enabled bridging solutions, and 3) to offer routes to market and strategic approaches at both industrial and public actor levels to drive adoption at scale of the proposed solutions and associated care models (see Figure 1 below).

At the same time, CATCH will develop and deliver a program of training to create a cadre of 8 PhD students who, after the training, will be able to implement the required solutions in industry, clinical, advocacy, and academic environments. This paper focuses on the research element of the CATCH program. For further detail regarding the training elements of the program, please see Mountford et al, 2017 [13]. The students' Individual Research Projects (IRPs) will address all the key elements of the objectives. The combined IRPs will drive a new interdisciplinary approach to technology-enabled post-diagnosis rehabilitative solutions and their evaluation and will enhance adoption by healthcare providers, patients, and society.

## Methods

Three work packages have been proposed to achieve the three sub-objectives defined for the study and the PhD student training.

### Work Package 1: Understand the Nature of the Problem (Sub-Objective 1)

The first sub-objective consists in understanding, from a patient perspective, the nature of the physical and psychological gap that must be bridged to enable and achieve physical activity. To achieve this, the PhD students will perform the following tasks:

Task 1: An ethnographic analysis of the current care pathway from the patient perspective (PhD student 1). The PhD student will review patient care pathways in cancer treatment literature, including the latest publications, prior to conducting an ethnographic study of the patient care pathways for both men and women. The student will employ ethnographic research methods drawn from the discipline of anthropology [14]—the study of the development of human societies and culture [15]—which is known to more effectively capture patient insights [16] and will:

1. Review patient care pathways in cancer treatment literature.
2. Conduct an ethnographic study of the patient care pathway by observing and interacting with cancer patients to comprehend the lived-experience of particular care pathways.
3. Analyze data to develop a detailed patient pathway that meets the needs of both sexes for all types of cancer.
4. Identify elements of the care pathway where there are opportunities for technology-enabled post-diagnosis rehabilitation.

Task 2: A quantification of health habits and needs of people affected by cancer to improve their QoL through physical activity (PhD student 2). By engaging in ethnographic research with cancer patients involved in various forms of physical exercise, this will result in the design of a digital intervention to maximize the medical, physical, functional, and psychological effects of physical activity programs for men and women with cancer. The PhD student will:

1. Conduct a comprehensive literature review, including the latest publications, focusing on medical, physical, functional and psychological aspects of cancer treatment for both men and women.
2. Undertake qualitative research with male and female patients to identify factors crucial for the design of a digital intervention.
3. Develop guidelines for cancer patient populations regarding health and lifestyle, paying specific attention to physical activity and how to improve patient QoL during the phases of cancer treatment. These guidelines will be tested onsite at the Center of Sport at University of Seville.
4. Design a digital intervention to assist patient adherence to physical exercise regimes.

Task 3: Strategies for increasing mental well-being in patients with cancer (PhD student 3). The PhD student will investigate strategies for using digital health to reinforce and improve psycho-oncology interventions (addressing emotional and mental health issues associated with cancer). Focusing on behavioral and emotional support—increasing motivation and reducing asthenia and fatigue—the student will devise new strategies to improve patients’ psychological and physical well-being. The PhD student will perform the following tasks:

1. Review literature to include most recent publications on increasing motivation, supporting positive health behavior, and prior use of technology in mental health support, including a review of psycho-oncology projects that use new technologies.
2. Conduct qualitative research (interviews, focus-groups, workshops) with a diverse group of patients and caregivers with different cancers from both genders. This will provide a detailed and empathic understanding of cancer patients’ lived experiences.
3. Develop a motivational framework that maps behavioral and emotional strategies for diverse cancer patients and survivors. Based on the results, a new app will be designed that instantiates the framework which will then be tested to redefine the motivational framework.

### Work Package 2: Design and Test Technology-Enabled Bridging Solutions (Sub-Objective 2)

To achieve this sub-objective, three different technology solutions will be selected and explored to evaluate how they can be matched to specific cancer care scenarios.

Solution 1: Psychological solutions that use gamification and education to empower cancer patients experiencing difficulty regaining their former role in society (PhD student 4). For this study the student will:

1. Perform an up-to-date systematic scoping review of games, techniques, and previous cancer-specific digital solutions. Based on the obtained experience, a gamified application will be implemented.
2. Implement a first version of a social mobile app for prostate cancer patients and survivors.
3. Study and consult with oncology experts regarding the safety, usefulness, and accuracy and refine the app based on this consultation.
4. Evaluation of the app using an A/B based testing protocol with real patients.

Solution 2: Physical solutions, such as electrical stimulation, to help re-educate and strengthen muscle severely weakened as a result of aggressive cancer (PhD student 5). The PhD student will:

1. Conduct a study to understand the exercise rehabilitation needs of patients with breast or prostate cancer.
2. Develop an NMES-mediated hybrid training protocol that meets the specific needs of the two significant cancer survivor cohorts - prostate cancer and breast cancer. This will consist of an evaluation of the acute physiological and subjective effects of applying different variations of a hybrid NMES protocol in a cohort of the target population.
3. Conduct a pilot trial with a group of prostate and breast cancer survivors who are too de-conditioned to meaningfully benefit from standard physical exercise. The trial will require patients to undergo self-directed, home-based NMES training for a period of 8-16 weeks. Approximately 40 participants (equally gender balanced) will be recruited for the prospective trial. The program will be evaluated through clinical and functional outcomes and user experience.
4. Study and understand the potential application of the outputs in the context of care pathways for cancer patients, particularly how this technology can be integrated with drug therapy for cachexia.

Solution 3: Use of motion tracking solutions and interactive biofeedback to monitor performance and improve compliance and quality of patient engagement in rehabilitation exercise programs (PhD student 6). The PhD student will:

1. Conduct a literature review, including the latest publications, on cancer-specific, targeted rehabilitation exercise programs, focusing on the information gap between the patient, consultant, and physiotherapist and the patient’s ability to access information about their own care.
2. Gather data from cancer patients performing exercises in a clinical setting to inform the exercise classification model.
3. Develop exercise classification algorithms and a prototype app tailored specifically to each patient’s needs by their clinician. This would allow the patient to attain credible information on their rehabilitation progress, regardless of their geographical location.
4. Evaluate the prototype in clinical deployment, and then evaluate and rework it from a ‘technology and care’ model perspective.

### Work Package 3: Offer Routes to Market and Strategic Approaches (Sub-Objective 3)

The last objective aims to offer routes to market and strategic approaches to drive adoption at scale of the proposed solutions and associated care models at both an industry and public actor level.

To achieve this sub-objective, two different approaches are covered:

Approach 1: Supporting commercialization of technology- enabled cancer solutions through design thinking (PhD student 7). The PhD student will:

1. Conduct an up-to-date systematic review of Service Innovation and related literature, commercialization, and design literature complemented by interviews (cancer patients, researchers, clinicians) to understand how we can mediate between heterogeneous innovators and between internal and external surroundings.
2. Develop preliminary guidelines for how private firms can use design thinking (methods) to qualify the process of developing technology-enabled cancer care solutions (collaboration, innovation, diffusion, and commercialization).
3. Complete workshops with users and partners to assess whether design has strengthened the firms’ awareness of different external and internal challenges and how to handle them.

Approach 2: Qualifying private organizations’ commercialization efforts through stakeholder interactions (PhD student 8). The student will:

1. Conduct an up-to-date systematic review of Service Innovation and related literature, commercialization, and stakeholder literature on turning health solutions into commercial successes.
2. Study the context in which cancer care solutions are developed including interviews with private and public actors engaged in (Public-Private) Service Innovation.
3. Develop preliminary guidelines for how private firms can use stakeholder interactions to qualify commercialization efforts.
4. Pilot and evaluate the (Public-Private) Service Innovation guidelines and assess whether stakeholder interactions have strengthened private firms’ awareness of different challenges and how to handle them.

### Program Management and Ethos

Each sub-objective and its associated work package promote state of the art research in its area. All three work packages are concurrent and will cross-pollinate. Information from work package 1 will provide design input for work package 2 and context for work package 3. Work package 2 will provide case studies and data sources to inform Work Package 3. This will enable a truly interdisciplinary learning environment for the PhD students and a triangulated, defensible approach from an industry-relevance perspective. The tasks will be undertaken by PhD candidates from a range of scientific backgrounds and international locations, as well as across both genders. CATCH will include international, intersectoral secondments to promote interdisciplinary and intersectoral learning and communication, public engagement, and outreach with patients, clinicians, and policy-makers. Building on the core tasks outlined above, an end-to-end technology enabled cancer care research spectrum will be designed from problem definition, to solution design, to implementation and adoption. CATCH will consider the importance of technology not only at the treatment stage, but also during rehabilitation and follow up. Through network-wide events and training modules, the individual research outcomes will synergistically contribute to this complete model. Through diverse means—face-to-face contact in summer schools, electronic communication platforms (e.g. Podio), and physical embedding (secondments)—PhDs will work with non-academic partners to appreciate different perspectives, methods, and approaches. The non-academic sector partners are integral for the development and delivery of the program. Industry partners will recruit and train students, host site visits, host secondments, provide keynote speakers for summer schools, will sit on doctoral studies panels, and will run a transferrable skills module on innovating in an emerging market. Healthcare partners will recruit and host students, run a transferrable skills module on working with patient populations, lead dissemination efforts to clinical audiences, and provide a venue for students to see a clinical setting in practice during the Orientation conference. Patients will contribute as cohorts for studies and participate in the “working with patients” training module, thus contributing to user experience training. Clinicians from healthcare partners will assist in sourcing cohorts for trials, user-centered design, and membership of supervisory panels for care-led PhDs and attendance at clinician conference.

## Results

This program has now received funding under the European Union's Horizon 2020 research and innovation program under the Marie Sklodowska-Curie grant agreement No 722012. All eight students have now been recruited and have commenced their PhD programs.

## Discussion

The emerging Connected Health industry is in need of research that combines deep domain-specific expertise and a complementary understanding of how such expertise fits into this intersectoral, interdisciplinary ecosystem. Europe needs to keep up to date with international developments in healthcare (electronic health [eHealth], mobile health [mHealth], telemedicine), but currently, researchers are graduating with narrow mono-thematic, purely academic degrees that limit understanding of the range of solutions possible [17]. CATCH will provide a comprehensive training and research program by embracing all key elements—technical, social, and economic sciences—required to produce researchers and project outcomes that are capable of meeting existing and future needs in cancer rehabilitation. In doing so, CATCH also responds to a fundamental need for a new health care model [18] that can counterbalance the demographic and resource pressures faced by our healthcare systems.

## Abbreviations

eHealth: electronic health
IRP: Individual Research Projects
mHealth: mobile health
QoL: Quality of Life
CATCH: Cancer: Activating Technology for Connected Health
